# Neurotoxicologically Outcomes of Perinatal Chlordiazepoxide Exposure on the Fetal Prefrontal Cortex Cells in Rat Pup: Chlordiazepoxide Effects on Fetal Prefrontal Cortex

**DOI:** 10.31661/gmj.v14i.3649

**Published:** 2024-12-31

**Authors:** Yekta Parsa, Zahra Nadia Sharifi, Fariborz Ghaffarpasand, Tina Parsa, Mohammad-Reza Zarrindast, Ehsan Jangholi, Soheila Yadollah-Damavandi, Mohammad Hossein Aghazadeh, Farshad Shahi, Shabnam Movassaghi

**Affiliations:** ^1^ Young Researchers and Elite Club, Tehran Medical Sciences Branch, Islamic Azad University, Tehran, Iran; ^2^ Anatomical Sciences Department, Tehran Medical Sciences Branch, Islamic Azad University, Tehran Iran; ^3^ Research Center for Neuromodulation and Pain, NAB Pain Clinic, Shiraz University of Medical Sciences, Shiraz, Iran; ^4^ Department of Pharmacology, Medical Genomics Research Center and School of Advanced Sciences in Medicine, Tehran University of Medical Sciences, Tehran, Iran; ^5^ Department of Neurosurgery, Tehran University of Medical Sciences, Tehran, Iran

**Keywords:** Chlordiazepoxide, Prefrontal Cortex, Perinatal Exposure, Neurotoxicity, Rat

## Abstract

**Background:**

Chlordiazepoxide is a benzodiazepine which is widely used as an anxiolytic, sedative and muscle-relaxant and its effects on neurodevelopment is yet to be identified. The aim of the current experimental study was to determine the effects of prenatal exposure to chlordiazepoxide on development of the prefrontal cortex (PFC).

**Materials and Methods:**

A total number of 9 pregnant Wister rats that were randomly assigned to three groups receiving standard rat food and drinking water ad libitum (n=3) or chlordiazepoxide (10 mg/kg) (n=3) and an equal volume of vehicle (0.9% NaCl) (n=3) intraperitoneal (i.p.) injection once daily from first to 21st day of gestation, respectively. At the end of the experiment, 14-day-old neonatal rat pups (n=8 per each group) were sacrificed and their PFC cells were extracted. Mitochondria were extracted from the PFC cells and their level of reactive oxygen species (ROS), protein density, Glutathione (GSH) content, mitochondrial membrane potential (MMP), swelling, cytochrome c release and ATP level was identified. We also performed the Nissl staining, DNA fragmentation assay and RNA extraction and real-time polymerase chain reaction (PCR) on PFC cells.

**Results:**

We found that isolated mitochondria from rat pups receiving chlordiazepoxide (E), had significantly higher ROS formation (P0.001), decreased GSH (P0.001), lower MMP (P0.001), higher mitochondrial swelling (P0.001), decreased ATP level (P0.001), increased cytochrome c release (P0.001) and higher Bax (P0.001), p53 (P0.001), cytochrome c (P0.001) and caspase 8 mRNAs (P0.001). The Nissle-stained neurons decreased while the apoptosis significantly increased (P0.001).

**Conclusions:**

The results of this in vivo study provide evidence regarding negative effects of prenatal exposure to chlordiazepoxide on PFC.

## Introduction

Chlordiazepoxide is a benzodiazepine which is widely used as an anxiolytic, sedative and muscle-relaxant. The biologically active metabolite of chlordiazepoxide is oxazepam which is produced through an enzymatic pathway. Chlordiazepoxide can easily cross the placenta and accumulate in the fetal circulation reaching three times higher than maternal serum levels [1][2]. Chlordiazepoxide connects to benzodiazepine allosteric sites which are parts of GABA receptor/ion-channel complex.

This results in increased binding of the inhibitory neurotransmitter GABA to its receptor leading to inhibitory effects of central nervous system (CNS) [3][4][5]. The American Congress of Obstetricians and Gynecologists categorizes chlordiazepoxide as class D which indicates positive evidence of human fetal risk based on adverse reaction data from investigational or marketing experience or studies in humans, but potential benefits may warrant use of the drug in pregnant women despite potential risks [6][7].

There is controversy regarding the teratogenic effects of prenatal exposure to chlordiazepoxide. It has been previously demonstrated that chlordiazepoxide administration resulted in avoidance behavior and decreased benzodiazepine receptor density in adult albino rats [3][8].

Several congenital anomalies have been reported to be associated with administration of chlordiazepoxide during the pregnancy including microcephaly, duodenal atresia and cardiovascular anomalies [9][10]. However, some recent studies have eliminated the risk of congenital anomalies with antenatal consumption of benzodiazepine especially diazepam and chlordiazepoxide [11]. Currently, data regarding the effects of prenatal fetal exposure to chlordiazepoxide on development of the cerebral cortex is scarce in the literature. Thus, the aim of the current experimental study was to determine the effects of prenatal exposure to chlordiazepoxide on development of the PFC.

## Materials and Methods

**Table:** Table1. Details of Primers (Forward and Reverse) for Target Genes Used for RT-PCR Analysis

**Gene**	**Primers sequence (5′-3′)**	**Product length**
Caspase 8	F: AGCAGCCTATGCCACCTAGT R: GCTGTAACCTGTCGCCGAG	261 bp
Tp53	F: GGTACCGTATGAGCCACCTG R: AACCTCAAAGCTGTCCCGTC	166 bp
Bax	F: CCAAGAAGCTGAGCGAGTGT R: CCCAGTTGAAGTTGCCGTCT	156 bp
Bcl-2	F: TCTTTGAGTTCGGTGGGGTC R: GTTCCACAAAGGCATCCCAG	153 bp
Cytochrome c	F: CCAGGCTGCTGGATTCTCTT R: GGTCTGCCCTTTCTCCCTTC	158 bp
GAPDH	F: AAGTTCAACGGCACAGTCAAGG R: CATACTCAGCACCAGCATCACC	121 bp

**Figure-1:**
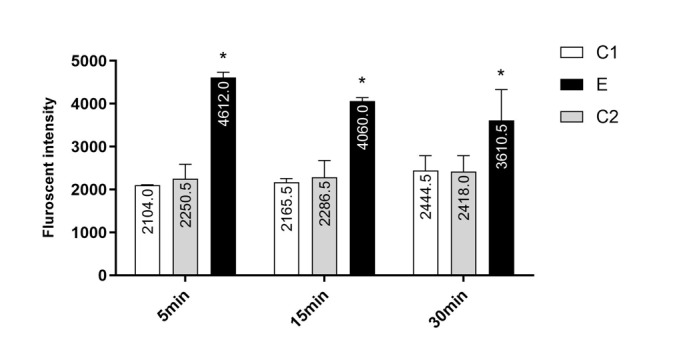
Figure 1. ROS formation in isolated prefrontal cortex mitochondria. ROS formation was evaluated fluorometrically using DCF-DA in the time intervals (5, 10 and 30 min) following isolation of rat brain mitochondria in all groups. The data was presented as mean ± SD (n=3). *P<0.001 compared with control groups.

**Figure-2:**
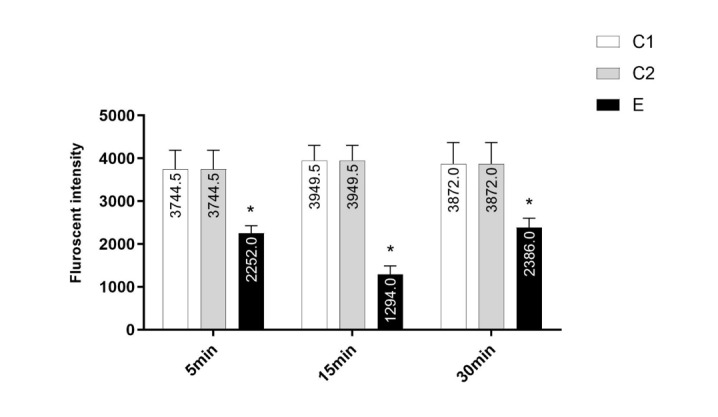
Figure 2. MMP decrease in the prefrontal cortex mitochondria isolated from pups. MMP was measured by rhodamine 123 as described in materials and methods. The data was presented as mean ± SD (n=3). *P<0.001 compared with control groups.

### 1. Animals

In this experimental study nulliparous female Wistar rats (170-195 g) were obtained from the Pasteur Institute (Karaj, Iran). The rats were housed in standard cages at 25±2°C and 12:12-hrs light-darkness cycle with free access to water and food ad libitum. Two females were placed overnight with a proven fertile male in a breeding cage. Copulation was ascertained on the next morning by finding sperm in vaginal smears or vaginal plug, and this was counted as first day of pregnancy. The female rats with positive vaginal smears/plug were weighed and randomly assigned to study.

All experimental procedures used in the current study were performed in accordance with the ARRIVE ethical guidelines, and were reviewed and approved by the Institutional Animal Care and Ethics Committee of Islamic Azad University, Tehran Medical Sciences Branch, Tehran, Iran. The experimentation adhered to the Guide for the Care and Use of Laboratory Animals (NIH Publication No. 86-23, 1985 edition).

### 2. Study Design

Nine pregnant Wistar rats were randomly divided into three groups (n=3): the control group received standard rat food and drinking water ad libitum. Rats in experimental and vehicle groups received Chlordiazepoxide (Sigma-Aldrich, Germany) (10 mg/kg) [2] and an equal volume of vehicle (0.9% NaCl) intraperitoneal (i.p.) injection once daily from first to 21th day gestation, respectively. At the end of the experiment, fourteen-day-old neonatal rat pups (n=8 per each group) that delivered by rats of control, chlordiazepoxide-treated, and vehicle groups were assigned into C1, E, and C2 groups, respectively.

### 3. Tissue Collection and Sample Preparation

All the pups were anesthetized with an intraperitoneal injection of sodium pentobarbital (40 mg/kg), and xylazine (10 mg/kg) 5-min before being perfused. For molecular analysis, pups received transcardiac perfusion of normal saline (pH: 7.4) followed by decapitation.

The olfactory bulb and cerebellum were removed and were not used in the current study but used for educational purposes; the brain hemispheres were snap-frozen in liquid nitrogen and stored at −80°C. For immunohistochemical analysis, animals were perfused with PBS followed by perfusion of 4% paraformaldehyde at pH 7.4 and the brains were postfixed at 4°C for 3 days, embedded in paraffin. Coronal sections through the prefrontal cortex were made according to the figures 9 and 11 in the rat brain in stereotaxic coordinates [12] and further processed for histological staining.

### 4. Isolation of Mitochondria

Mitochondria were prepared from the PFC (n=3 rats per group) using differential centrifugation [13]. The desired tissues were removed and minced with a small scissor in a cold mannitol solution containing 0.225 M D-mannitol, 75 mM sucrose, and 0.2 mM EDTA. The minced desired tissues were gently homogenized in a glass homogenizer with a Teflon pestle and then centrifuged at 1000×g for 10 min at 4°C to remove the nuclei, unbroken cells, and other non-subcellular debris. The supernatants were centrifuged at 10,000×g for 10 min. The dark packed lower layer (mitochondrial fraction) was resuspended in the mannitol solution and re-centrifuged twice at 10,000×g for 10 min. Mitochondrial sediments were suspended in Tris solution containing 0.05 M Tris-HCl buffer (PH=7.4), 0.25 M sucrose, 20 mM KCl, 2.0 mM MgCl2, and 1.0 mM Na2HPO4 at 4 ºC before the assay. Aliquots of the suspension were used to determine the multi-parameters of oxidative stress. All tests were performed three times.

### 4.1. Protein Concentration

Protein concentrations were determined by the Coomassie blue protein-binding method as explained by Bradford et al. [14] The isolation of mitochondria was confirmed by the measurement of succinate dehydrogenase [15]. Mitochondria were prepared freshly for each experiment and used within 4-hour of isolation, and all steps were strictly operated on ice to guarantee the isolation of high-quality mitochondrial preparation.

### 4.2. Quantification of Mitochondrial ROS Level

Isolated mitochondria were placed in respiration buffer containing 0.32 mM sucrose, 10 mM Tris, 20 mM MOPS, 50 μM EGTA, 0.5 mM MgCl2, 0.1 mM KH2PO4 and 5 mM sodium succinate [16]. Following this step, a sample was taken and diacetyldichlorofluorescein (DCFH) was added (final concentration, 10 μM) to the mitochondria and was then incubated for 15 min. The fluorescence intensity of dichlorofluorescein (DCF) was measured using Shimadzu RF-5000U fluorescence spectrophotometer at an excitation wavelength of 488 nm and an emission wavelength of 527 nm.

### 4.3. Measurement of Glutathione (GSH) Content

The mitochondrial fractions were added into 0.1 mol/L phosphate buffers and 0.04% DTNB [5,5-dithio-bis-(2-nitrobenzoic acid)] in a total volume of 3.0 mL (pH=7.4). The developed yellow color was read at 412 nm using a spectrophotometer (UV-1601 PC, Shimadzu, Japan). GSH content was expressed as µg/mg protein [17].

### 4.4. Mitochondrial Membrane Potential (MMP) Assay

The mitochondrial uptake of the fluorescent cationic dye, rhodamine123, has been used for the determination of mitochondrial membrane potential. The mitochondrial suspensions (500 µg protein /mL) were incubated with 10 RM of rhodamine123 in the MMP assay buffer (220 mM sucrose, 68 mM D-mannitol, 10 mM KCl, 5 mM KH2PO4, 2 mM MgCl2, 50 μM EGTA, 5 mM sodium succinate, 10 mM HEPES, 2 μM Rotenone).

The fluorescence was measured using Schimadzou RF-5000U fluorescence spectrophotometer at the excitation and emission wavelength of 490 nm and 535 nm, respectively [18]. The capacity of mitochondria to uptake the rhodamine123 was calculated as the difference (between control and treated mitochondria) in rhodamine123 fluorescence. Data was shown as the percentage of mitochondrial membrane potential collapse (%∆Ψm) in all treated mitochondria groups.

### 4.5. Determination of Mitochondrial Swelling

Isolated mitochondria were suspended in swelling buffer (70 mM sucrose, 230 mM mannitol, 3 mM HEPES, 2 mM Tris-phosphate, 5 mM succinate and 1 RM of rotenone) [19]. The absorbance was measured at 540 nm at 10 min time intervals with an ELISA reader (Tecan, Rainbow Thermo, Austria). A decrease in absorbance indicates an increase in mitochondrial swelling.

### 4.6. Cytochrome C Release Assay

The concentration of cytochrome c was determined through using the Quantikine Rat/Mouse Cytochrome c ELIZA kit. Briefly, a monoclonal antibody specific for rat/mouse cytochrome c was pre-coated onto the microplate. Seventy-five μL of the conjugate (containing monoclonal antibody specific for cytochrome c conjugated to horseradish peroxidase) and 50 μL of control and test group were added to each well of the microplate. One microgram of protein from each supernatant fraction was added to the sample wells. All of the standards, controls and test were added to two wells of the microplate. After 2 h of incubation, the substrate solution (100 μL) was added to each well and incubated for 30 min. After 100 μL of the stop solution was added to each well; the optical density of each well was determined by the aforementioned microplate spectrophotometer set to 450 nm.

### 4.7. Assay of ATP Level

The ATP levels were measured using Luciferin/Luciferase Enzyme system [20]. Bioluminescence intensity was measured using Sirius tube luminometer (Berthold Detection System, Germany). ATP level was expressed as µg/mg protein

### 5. Nissl Staining

Nissl staining was performed as described previously [21]. Briefly, the 10-μm sections were hydrated in 1% toluidine blue at 50 °C for 20 min. After rinsing with double distilled water, they were dehydrated and mounted with permount. After rinsing with double distilled water, the sections were dehydrated in increasing concentrations of ethanol and cleared in xylene, then mounted with Permount cover slip and observed under a light microscope. Cells that contained Nissl substance in the cytoplasm, loose chromatin, and prominent nucleoli were considered normal neurons, and damaged neurons were identified by the loss of Nissl substance, cavitation around the nucleus and by the presence of pyknotic nuclei.

### 6. DNA Fragmentation Assay

The TUNEL technique was applied to determine the extent of neuronal cell death in tissue sections. Therefore, the commercially available Fluorescein In Situ Cell Death Detection Kit (Sigma-Aldrich Co., USA) was used according to the manufacturer's instructions. In brief, slides were dried for 30 min followed by fixation in 10% formalin solution at RT. After washing in PBS, sections were incubated in an ice-cold ethanol-acetic acid solution (3:1), washed with PBS and incubated with 3% Triton X-100 solution for 60 min at RT for permeabilization. Slides were then incubated with the TdT-enzyme in reaction buffer containing fluorescein-dUTP for 90 min at 37°C. Negative control was performed using only the reaction buffer without TdT enzyme. Positive controls were carried out by digesting with 500 U/ml DNase grade I solution. To preserve cells for comparison, slices were covered with Vectashield® mounting medium containing 4',6'-diamino-2-phenylindole (DAPI). All samples were evaluated immediately after staining using an ''Axioskop 40'' fluorescence microscope (Zeiss, Germany) at 460 nm for DAPI and 520 nm for TUNEL Five visual fields (0.6 mm2) of the cerebral cortex were photographed in each section. The number of staining cells in each field was counted at higher magnification (×40). The data were represented as the number of cells per high-power field.

### 7. RNA Extraction and Real-Time Polymerase Chain Reaction (PCR)

Total RNA was extracted utilizing TRIzol reagent according to manual’s protocol. After extraction, all RNA samples were treated with DNase I. Total cDNA was synthesized by cDNA synthesis kit according to the kit’s instruction. Primers were designed specifically to amplify cDNA of caspase 8, Tp53, bax, bcl2, cytochrome c and Glyceraldehyde 3-phosphate dehydrogenase (GAPDH, as an internal control for normalization).

The target genes and the specific primers are shown in Table-1. The resulting cDNA amplification was performed using the 7500 Fast Real-Time System (Applied Biosystems, USA) in conjunction with the SYBR Premix Ex Taq TM II kit. The PCR reactions were programmed as follows: Holding Stage: 95°C, 30 sec. Cycling Stage: denaturing step: 95°C, 5 sec, followed by annealing/amplification step 60°C, 30 sec (Number of Cycles: 40). The relative quantification of gene expression level was analyzed by the 2- ΔΔCt method. The level change in target gene cDNA relative to the GAPDH internal control was determined by:

Level change= 2- ΔΔCt, Where ΔΔCt= (Ct target gene- Ct GAPDH)-(Ct control-Ct GAPDH)

### 8. Statistical Analysis

To have 80% power to detect at least 5% difference between the study groups regarding the main study outcomes, we proposed to include at least 3 rats in each study group. As this was a small sample size, we ran a power calculation at the end of the study to ensure the reliability of the results. All data are presented as mean±SD. Five visual fields (0.25 mm× 0.25 mm) of the prefrontal cortex were photographed in each section. The positive pyramidal cells of Nissl and TUNEL in the unit area were counted in a high-power field (×400) by using Imaging-Pro-Plus (LEICA DMLB, Germany) software and the cell count obtained from the same group of the animal were averaged and expressed as number/mm2.

Microscopic examinations were made by a single pathologist who was unaware of the characteristics or treatment of the animals. Statistical comparisons were analyzed by one-way analysis of variance (ANOVA) followed by Tukey’s posttest for differences among the groups using GraphPad Prism software version 5.0 (GraphPad Software, USA). A 2-sided p-value of less than 0.05 was considered statistically significant.

## Results

**Figure-3:**
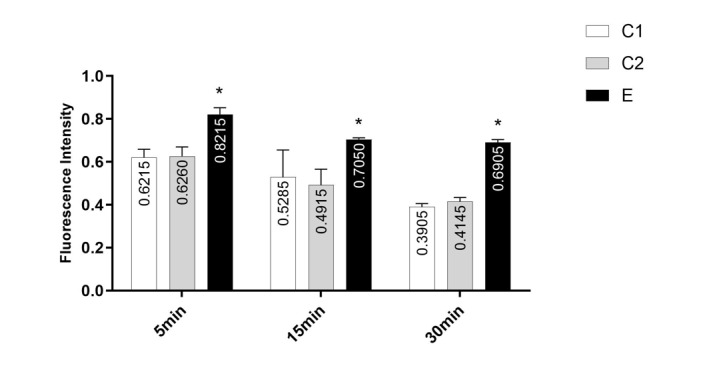
Figure 3. Progressive mitochondrial swelling in the prefrontal cortex mitochondria isolated from all pups. Mitochondrial swelling was measured through the determination of absorbance at 530 nm. The data was presented as mean ± SD (n=3). *P<0.001 compared with control groups.

**Figure-4:**
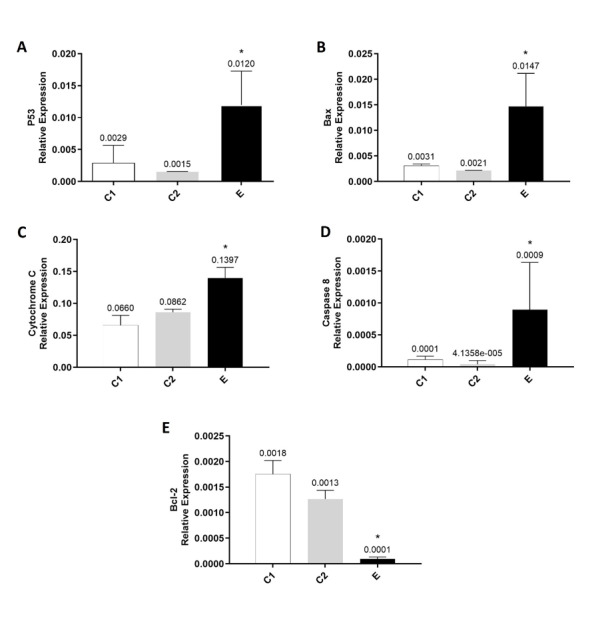
Figure 4. Quantitative real-time polymerase chain reaction (PCR) analysis of relative expression of mRNAs in the prefrontal cortex. The p53 (A), bax (B), cytochrome c (C), caspase 8 (D) and bcl-2 (E) mRNAs in the prefrontal cortex. Results are presented as means ± SD of the normalized PCR product concentrations for each dilution step (n=3). *P<0.01 compared with control groups.

**Figure-5:**
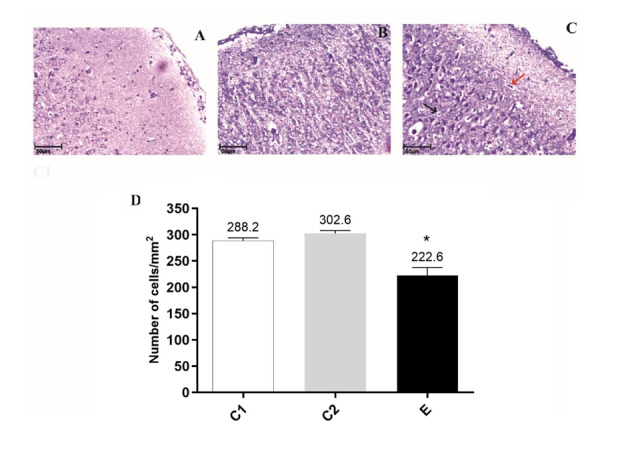
Figure 5. Nissl standing of pup’s prefrontal cortex. The prefrontal cortex of pups in the C1 and C2 groups (A and B) did not contain any damaged neurons. However, the cortex of pups in the E group(C) was characterized by more Nissl stain neurons (black arrow) than control groups, and markedly contained shrunken, intensely-stained and dystrophic neurons (red arrow). As showed in the graph (D), maternal chlordiazepoxide administration significantly induced neuronal cell loss in the prefrontal cortex. Data was represented as mean ± SD (n=3). Scale bars 50μm, magnification ×400. *P<0.001 vs. control groups.

**Figure-6:**
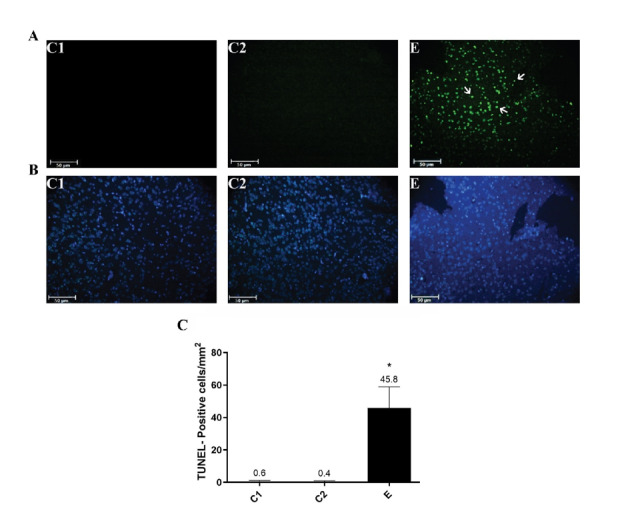
Figure 6. Effects of maternal chlordiazepoxide administration on induced apoptotic neurodegeneration. Representative photomicrographs of TUNEL staining and cell counting. A: Representative images of the TUNEL-positive cell (arrows) were obtained from sections prepared from the pups in all groups. B: The overall cellular morphology of the TUNEL sections is revealed by DAPI nuclear stain. C: Maternal chlordiazepoxide administration significantly increased the TUNEL-positive cell compare to C1 and C2 groups. Data was represented as mean±SD (n=3). Scale bars 50 μm, magnification ×400. *P<0.005 vs. control groups.

### Chlordiazepoxide Induced ROS Formation in Isolated Prefrontal Cortex Mitochondria

The formation of ROS in the isolated prefrontal cortex mitochondria from C1 and C2 pups was significantly lower than in group E (P<0.001). There was no significant difference between C1 and C2 pups (2238±181.5 vs. 2318±88.17, P=0.99). ROS formation was measured in the time intervals (5, 15 and 30 min) following isolation of prefrontal cortex mitochondria in all groups (Figure-1).

### Effect of Chlordiazepoxide on GSH Content in Isolated Prefrontal Cortex Mitochondria

The content of GSH in isolated prefrontal cortex mitochondria of pups in the E group significantly decreased when compared to pups in the C1 and C2 groups (P<0.001, Figure-S1). Furthermore, GSH content was not statistically different between groups C1 and C2 (0.62±0.02 vs. 0.64±0.01, P=0.35).

### Effect of Chlordiazepoxide on the MMP in Isolated Prefrontal Cortex Mitochondria

The MMP is a highly sensitive indicator of the mitochondrial inner membrane condition and was measured by Rhodamine 123 redistribution. The MMP in isolated prefrontal cortex mitochondria of pups in C1 and C2 groups was higher than E group (P<0.001). As indicated in Figure-2, the mother treatment with chlordiazepoxide has induced noticeable decrease in the MMP in prefrontal cortex from rat puppies compared to puppies born from females that were not treated.

### Effect of Chlordiazepoxide on Mitochondrial Swelling

Mitochondrial swelling in the group E was significantly higher than that of the control groups (P<0.001) as demonstrated in Figure-3. However, there was no significant difference between group C1 and C2 (0.51±0.11 vs. 0.51±0.1, P=0.99).

### Effect of Chlordiazepoxide on Mitochondrial ATP Level

The mean mitochondrial ATP level in the pups’ prefrontal cortex of groups C1 and C2 was 97.33±3.05 and 95±2, respectively (P=0.47). Maternal chlordiazepoxide administration showed a significant (P<0.001) decrease in the ATP level of mitochondrial isolated from prefrontal cortex when compared to group C1 and C2 (Figure-S2).

### Chlordiazepoxide induced Cytochrome C Release in Isolated Brain Mitochondria

The mean cytochrome c release, and the endpoint of mitochondrial toxicity in the pups’ prefrontal cortex of group C1 and C2 was 132.3±1.52 and 130.3±1.52, respectively (P=0.52). The mean cytochrome c release was significantly increased in the E group when compared to groups C1 and C2 (P<0.001, Figure-S3).

### The Effect of Chlordiazepoxide on the Expression of Bax, Bcl-2, Cytochrome C, P53 and Caspase 8 mRNAs in the Prefrontal Cortex

Relative expression of Bax, p53, cytochrome c and caspase 8 mRNAs in the prefrontal cortex of C1 and C2 groups were lower than those of the E group, although relative expression of bcl-2 mRNA was significantly higher than those of the E group (Figure-4; P<0.01). As indicated in Figure-4, the treatment of rats with chlordiazepoxide significantly increased the relative expression of pro-apoptotic mRNAs, such as Bax, p53, cytochrome c and caspase 8 (Figure-4 A, B, D and E; P<0.01), and decreased the relative expression of Bcl-2 in group E compared to groups C1 and C2 (Figure-4 C and E; P<0.01).

### Effect of Chlordiazepoxide on Caspase-3 Activity in the Prefrontal Cortex

Caspase-3 activity was evaluated by spectrophotometer. The higher absorbance value indicated higher caspase-3 activity and therefore higher incidence of apoptosis. Caspase-3 activity for the control groups was lower than that of group E (0.023±0.015 and 0.024±0.005 vs. 0.563±0.086, P<0.001, respectively). Maternal chlordiazepoxide administration significantly induced caspase-3 activity in the prefrontal cortex of pups in the E group (Figure-S4).

### Administration of Chlordiazepoxide Induced neuron Loss in the Prefrontal Cortex of Pups

Neuronal cell loss was examined by using a cresyl violet stain. Sections of the prefrontal cortex of pups revealed that neonate of the maternal Chlordiazepoxide-treated induces loss of granular neurons (Figure-5A), in contrast to C1 neonates (Figure-5B), or to C2 neonates (Figure-5C), which showed no degeneration in the same area. Indeed, the number of Nissl-stained cells was significantly reduced (Figure-5D, P<0.001) in E neonates than in C1 and C2 neonates (114.6±6.5 neurons for E vs. 288.2±6.05 and 302.6±5.41 neurons for C1 and C2, respectively).

### The Apoptogenic Effect of Chlordiazepoxide on the Prefrontal Cortex of Neonates

Apoptotic cell death was determined using TUNEL staining, and the results are presented in Figure-6. The prefrontal cortex staining of C1 and C2 neonates did not show obvious TUNEL-positive cells (Figure-6A and B). However, TUNEL-positive cells showing shrunken cell bodies and green fluorescent signal were detected in the prefrontal cortex of E group neonates (Figure-6A and B). Indeed, the administration of Chlordiazepoxide during pregnancy significantly increased the number of TUNEL-positive cells in the prefrontal cortex of pups compared to the control groups (Figure-6C, P<0.05).

## Discussion

Few data are available regarding the teratogenic effects of chlordiazepoxide. There is controversy regarding the teratogenic effects of prenatal exposure to chlordiazepoxide. Bellantuono et al. [11] performed a meta-analysis in order to investigate the teratogenic effects of benzodiazepines. They have reported that the published data within the previous 10 years does not point out an absoulute contraindication for prescription of benzodiazepines during the first trimester of the pregnancy [11].

Most of the studies on the subject suffer from methodological shortcomings such as recall bias, presence of several confounding factors and missing data on malformations in aborted fetuses [3][2][4][1].

There are also limited experimental studies on the issue most of them belonging to the old literature [22][4]. Thus, we conducted this experimental in vivo study in order to investigate the role of prenatal exposure to chlordiazepoxide on development of the PFC. We found that there was excessive mitochondrial dysfunction in PFC due to oxidative stress in those that were exposed to chlordiazepoxide. We also observed that intrauterine exposure to chlordiazepoxide was associated with excessive apoptosis and cell death in PFC. To the best of our knowledge; this is among the only available data in the literature on the effects of prenatal benzodiazepines in PFC development.

The advantage of the current study was its methodology. We investigated the effects of intrauterine chlordiazepoxide exposure on PFC cells of the rat pups by molecular and cellular mechanisms instead of clinical parameters. The development and function of the PFC is highly energy demanding characterized by high number of mitochondria and higher expression of mitochondria proteins messenger RNA (mRNA) [23]. In addition, it has been demonstrated that oxidative stress is an important indicative of mitochondrial injury and dysfunction [24] determined by increased intracellular levels of ROS and decreased GSH [25]. Increased production of ROS or decreased ability of mitochondria to neutralize the ROS results in oxidative stress of the cell elements such as DNA (leading to apoptosis and cell death), proteins (leading to increased carboxylate protein content) and lipids (cell membrane disruption leading to mitochondria and cell swelling) [26]. In the current study we measured the level of ROS, the GSH, mitochondrial protein content and swelling as markers of mitochondrial oxidative stress and injury [27].

We found that intrauterine exposure of rat pups to chlordiazepoxide resulted in increased ROS and decreased GSH resulting in increased oxidized intracellular protein content and mitochondrial injury determine by its swelling. We also observed that prenatal chlordiazepoxide exposure was associated with decreased mitochondrial function measured by decreased ATP level and increased MMP in PFC. Mitochondrial function, a key indicator of cell health, can be assessed by monitoring changes in MMP which is a sensitive indicator of the mitochondrial inner membrane [28]. A recent similar study by Dinarvand et al. [2] demonstrated that prenatal exposure to chlordiazepoxide is associated with increased neuronal damage in the hippocampus of neonatal Wistar rats.

The ultimate result of mitochondrial and cell injury as result of oxidative stress is the apoptosis and cell death. During the apoptosis, several protein families interact with each other resulting in activation of proteolytic protein families, the most important of which is the caspase family. Although several pathways have been recognized and described for apoptosis, the mitochondrial pathways are the most important one in human CNS [29]. One of the earliest events in neuronal hypoxia and injury is the inhibition of mitochondrial cytochrome oxidase activity [30]. In other words, hypoxia and oxidase stress of the cell results in decreased cytochrome c activity guiding the cell toward mitochondrial apoptotic pathway [31][29].

Furthermore, it has been well demonstrated that Bcl-2, Bax and p53 are potent neuroprotective and neurotrophic proteins with anti-apoptotic activities [31]. In, the current study, we observed a decrease in expression of Bcl-2, Bax, p53, cytochrome c and caspase 3 activity in PFC of the rat pups receiving intrauterine chlordiazepoxide.

This clearly demonstrates that prenatal chlordiazepoxide exposure resulted in oxidative stress of the PFC mitochondria starting the apoptotic pathways and cell death. We further evaluated the PFC of the chlordiazepoxide-treated rat pups regarding apoptosis and cell death.

We clearly demonstrated that there was severe neuronal loss and apoptotic activity in PFC of this population determined by cresyl violet staining and TUNEL-positive cells, respectively. To summarize all these molecular and cellular findings, intrauterine exposure to chlordiazepoxide leads to excessive oxidative stress of the PFC neurons and mitochondrial injury. This results in activation of mitochondrial apoptotic pathways leading to cell death and PFC atrophy.

We measured the variables related to isolated mitochondria of the PFC such as MMP, GSH, ATP level, protein density, ROS level and the swelling and the cytochrome c release. It has been previously demonstrated that the morphology of the mitochondria of PFC in primates brain correlated with cognitive function and working memory [32]. In addition, Kar et al. [33] demonstrated the important role of mitochondrial mRNAs in neuronal physiology and animal behavior in transgenic mice expressing the cytochrome c oxidase IV in PFC. Reichel et al. [34] demonstrated that depletion of the GABAergic interneurons in PFC and hippocampus in animal model of rat is associated with behavioral changes such as sensory processing, anxiety, hyperactivity, cognition dysfunction and acquisition of a spatial memory [34]. taking into account, the results of the current study, it could be concluded that prenatal exposure to chlordiazepoxide will result in depletion of PFC and amygdale neurons leading to behavioral changes and cognitive and memory dysfunction.

All the discussed issues were the cellular processes and pathways. But the clinical outcome should be also discussed. Previously, in two distinct animal studies, Avnimelech-Gigus et al. [3] and Gavish et al. [4] demonstrated that prenatal exposure to chlordiazepoxide is associated with decreased cerebral and cerebellar benzodiazepine receptors with sustained receptor affinity. This change in the number of receptors was associated with avoidance behaviors in chlordiazepoxide exposed rat pups [3].

These findings postulated a possible link between the prenatal chlordiazepoxide exposure and the development of PFC which plays an important role in cognitive behaviors [35][36]. In the current study we have demonstrated that prenatal exposure to the chlordiazepoxide is associated with several developmental deficits in PFC.

We note some limitations to our study. First, we did not measure the serum, peritoneal and fetal levels of chlordiazepoxide in both the rat mothers and the pups.

Thus, the ultimate concentration of the chlordiazepoxide leading to different anomalies cannot be discussed. However the prescribed concentrations were the same and we assume that all the animals had the same pharmacokinetics leading to similar ultimate serum and fetal levels of the drug. The other limitation was that we did not evaluate the changes in development of the amygdale as the main associated circuitry of the PFC [37][38]. This was because of limited sources for research support.

Probably future studies including the amygdale are warranted to shed light on the mechanism of the action. We note that the sample size was small and we have included a limited number of rats in each study group.

This was because of sophisticated laboratory methods being utilized in the current study along with our limited source of funding. However, the study have appropriate power to detect the differences between the groups regarding the primary and secondary outcomes. In addition, this study tried to detect the outcomes through a histopathological point of view rather that statistical one.

The final limitation is that we did not evaluate the behavioral changes of the rat pups in three distinct study groups, as we had to sacrifice the rates for molecular and laboratory investigations.

This is a basic study addressing the molecular and histological changes and the clinical application have not been evaluated as mentioned above. However, this basic study addresses an important clinical clue to limit the use of chlordiazepoxide during the pregnancy in order to avoid neurodevelopmental disorders. Further clinical study is required to complete the scenario of intrauterine exposure to chlordiazepoxide in regards to neurodevelopment.

## Conclusion

In conclusion, the results of this experimental study clearly demonstrates that prenatal exposure to chlordiazepoxide is associated with developmental anomalies of the PFC determined by higher ROS formation, decreased GSH, lower MMP, higher mitochondrial swelling, decreased ATP level, increased cytochrome c release and higher Bax, p53, cytochrome c and caspase 8 mRNAs. Further clinical studies are required to elucidate the issue.

## Conflict of Interest

There isn’t any conflict of interest to be declared regarding the manuscript.

## References

[R1] Iqbal MM, Aneja A, Fremont WP (2003). Effects of chlordiazepoxide (Librium) during pregnancy and
lactation. Conn Med.

[R2] Dinarvand A, Hashemi M, Dinarvand R, Movassaghi S, Jafarinia M (2022). The Effect of Chlordiazepoxide Consumption on the Hippocampus of
Neonatal Rats During Pregnancy. Galen Medical Journal.

[R3] Avnimelech-Gigus N, Feldon J, Tanne Z, Gavish M (1986). The effects of prenatal chlordiazepoxide administration on
avoidance behavior and benzodiazepine receptor density in adult albino rats. Eur J Pharmacol.

[R4] Gavish M, Avnimelech-Gigus N, Feldon J, Myslobodsky M (1985). Prenatal chlordiazepoxide effects on metrazol seizures and
benzodiazepine receptors density in adult albino rats. Life Sci.

[R5] Mohammadkhani M, Gholami D, Riazi G (2024). The effects of chronic morphine administration on spatial memory
and microtubule dynamicity in male mice's brain. IBRO Neurosci Rep.

[R6] Armstrong C (2008). ACOG guidelines on psychiatric medication use during pregnancy
and lactation. American Family Physician.

[R7] Singsai K, Saksit N, Chaikhumwang P (2024). Brain acetylcholinesterase activity and the protective effect of
Gac fruit on scopolamine-induced memory impairment in adult zebrafish. IBRO Neurosci Rep.

[R8] Grigoriadis S, Alibrahim A, Mansfield JK, Sullovey A, Robinson GE (2022). Hypnotic benzodiazepine receptor agonist exposure during
pregnancy and the risk of congenital malformations and other adverse
pregnancy outcomes: A systematic review and meta-analysis. Acta Psychiatr Scand.

[R9] Hartz SC, Heinonen OP, Shapiro S, Siskind V, Slone D (1975). Antenatal exposure to meprobamate and chlordiazepoxide in
relation to malformations, mental development, and childhood mortality. N Engl J Med.

[R10] Milkovich L, van den (1974). Effects of prenatal meprobamate and chlordiazepoxide
hydrochloride on human embryonic and fetal development. N Engl J Med.

[R11] Bellantuono C, Tofani S, Di Sciascio, Santone G (2013). Benzodiazepine exposure in pregnancy and risk of major
malformations: a critical overview. Gen Hosp Psychiatry.

[R12] Paxinos G, Watson C (1986).

[R13] Ghazi-Khansari M, Mohammadi-Bardbori A, Hosseini MJ (2006). Using Janus green B to study paraquat toxicity in rat liver
mitochondria: role of ACE inhibitors (thiol and nonthiol ACEi). Ann N Y Acad Sci.

[R14] Bradford MM (1976). A rapid and sensitive method for the quantitation of microgram
quantities of protein utilizing the principle of protein-dye binding. Anal Biochem.

[R15] Fortunato F, Deng X, Gates LK, McClain CJ, Bimmler D, Graf R (2006). Pancreatic response to endotoxin after chronic alcohol exposure:
switch from apoptosis to necrosis. Am J Physiol Gastrointest Liver Physiol.

[R16] Gao X, Zheng CY, Yang L, Tang XC, Zhang HY (2009). Huperzine A protects isolated rat brain mitochondria against
beta-amyloid peptide. Free Radic Biol Med.

[R17] Sadegh C, Schreck RP (2003). The spectroscopic determination of aqueous sulfite using Ellman’s
reagent. MURJ.

[R18] Hosseini MJ, Shaki F, Ghazi-Khansari M, Pourahmad J (2013). Toxicity of vanadium on isolated rat liver mitochondria: a new
mechanistic approach. Metallomics.

[R19] Zhao Y, Ye L, Liu H, Xia Q, Zhang Y, Yang X (2010). Vanadium compounds induced mitochondria permeability transition
pore (PTP) opening related to oxidative stress. J Inorg Biochem.

[R20] Tafreshi N, Hosseinkhani S, Sadeghizadeh M, Sadeghi M, Ranjbar B, Naderi-Manesh H (2007). The influence of insertion of a critical residue (Arg356) in
structure and bioluminescence spectra of firefly luciferase. J Biol Chem.

[R21] Faghani M, Ejlali F, Sharifi ZN, Molladoost H, Movassaghi S (2016). The Neuroprotective Effect of Atorvastatin on Apoptosis of
Hippocampus Following Transient Global Ischemia/Reperfusion. Galen Medical Journal.

[R22] Laviola G, de Acetis, Bignami G, Alleva E (1991). Prenatal oxazepam enhances mouse maternal aggression in the
offspring, without modifying acute chlordiazepoxide effects. Neurotoxicol Teratol.

[R23] Chandrasekaran K, Stoll J, Giordano T, Atack JR, Matocha MF, Brady DR (1992). Differential expression of cytochrome oxidase (COX) genes in
different regions of monkey brain. J Neurosci Res.

[R24] Ansari MA, Scheff SW (2010). Oxidative stress in the progression of Alzheimer disease in the
frontal cortex. J Neuropathol Exp Neurol.

[R25] Gawryluk JW, Wang JF, Andreazza AC, Shao L, Young LT (2011). Decreased levels of glutathione, the major brain antioxidant, in
post-mortem prefrontal cortex from patients with psychiatric disorders. Int J Neuropsychopharmacol.

[R26] Lenaz G (2001). The mitochondrial production of reactive oxygen species:
mechanisms and implications in human pathology. IUBMB Life.

[R27] Andreazza AC, Shao L, Wang JF, Young LT (2010). Mitochondrial complex I activity and oxidative damage to
mitochondrial proteins in the prefrontal cortex of patients with bipolar
disorder. Arch Gen Psychiatry.

[R28] Sakamuru S, Attene-Ramos MS, Xia M (2016). Mitochondrial Membrane Potential Assay. High-Throughput Screening Assays in Toxicology.

[R29] Yuan J, Yankner BA (2000). Apoptosis in the nervous system. Nature.

[R30] Dong Y, Zhang W, Lai B, Luan WJ, Zhu YH, Zhao BQ (2012). Two free radical pathways mediate chemical hypoxia-induced
glutamate release in synaptosomes from the prefrontal cortex. Biochim Biophys Acta.

[R31] Jarskog LF, Gilmore JH, Glantz LA, Gable KL, German TT, Tong RI (2007). Caspase-3 activation in rat frontal cortex following treatment
with typical and atypical antipsychotics. Neuropsychopharmacology.

[R32] Hara Y, Yuk F, Puri R, Janssen WG, Rapp PR, Morrison JH (2014). Presynaptic mitochondrial morphology in monkey prefrontal cortex
correlates with working memory and is improved with estrogen treatment. Proc Natl Acad Sci U S A.

[R33] Kar AN, Sun CY, Reichard K, Gervasi NM, Pickel J, Nakazawa K (2014). Dysregulation of the axonal trafficking of nuclear-encoded
mitochondrial mRNA alters neuronal mitochondrial activity and mouse behavior. Dev Neurobiol.

[R34] Reichel JM, Nissel S, Rogel-Salazar G, Mederer A, Kafer K, Bedenk BT (2014). Distinct behavioral consequences of short-term and prolonged
GABAergic depletion in prefrontal cortex and dorsal hippocampus. Front Behav Neurosci.

[R35] Petrides M, Tomaiuolo F, Yeterian EH, Pandya DN (2012). The prefrontal cortex: comparative architectonic organization in
the human and the macaque monkey brains. Cortex.

[R36] Yoon JH, Grandelis A, Maddock RJ (2016). Dorsolateral Prefrontal Cortex GABA Concentration in Humans
Predicts Working Memory Load Processing Capacity. J Neurosci.

[R37] Perlman SB, Almeida JR, Kronhaus DM, Versace A, Labarbara EJ, Klein CR (2012). Amygdala activity and prefrontal cortex-amygdala effective
connectivity to emerging emotional faces distinguish remitted and depressed
mood states in bipolar disorder. Bipolar Disord.

[R38] Swartz JR, Carrasco M, Wiggins JL, Thomason ME, Monk CS (2014). Age-related changes in the structure and function of prefrontal
cortex-amygdala circuitry in children and adolescents: a multi-modal imaging
approach. Neuroimage.

